# Transcriptomics reveals age-related changes in ion transport–related factors in yak lungs

**DOI:** 10.3389/fvets.2024.1374794

**Published:** 2024-05-08

**Authors:** Xiating Xie, Yating Wei, Yan Cui, Qian Zhang, Hongqin Lu, Liang Chen, Junfeng He

**Affiliations:** ^1^College of Veterinary Medicine, Gansu Agricultural University, Lanzhou, Gansu, China; ^2^Laboratory Animal, Lanzhou Institute of Biological Products, Lanzhou, Gansu, China

**Keywords:** yak, lungs, transcriptomics, FOXI1, SLC12A2, KCNMA1

## Abstract

Yaks inhabit high-altitude, low-oxygen regions, where ion transport functions play a crucial role in maintaining intracellular and extracellular ionic balance and regulating pulmonary vascular tension. These functions affect pulmonary ventilation and blood flow rate, aiding tissue development and enhancing oxygen transfer efficiency, thus facilitating better adaptation to hypoxic environments. To investigate the regulatory mechanisms of ion transport-related factors on the growth and development of yak lungs, we employed RNA sequencing (RNA-seq)for sequencing the transcriptome in the lung tissues of neonatal (1-day-old), juvenile (1-year-old), and adult (4-year-old) yaks. We also performed differential gene expression and functional analyses. The results yielded 26 genes associated with ion transport, mainly enriched in the salivary and pancreatic secretion pathways. Finally, we used several methods including quantitative polymerase chain reaction (qRT-PCR), and Western blotting (WB), immunohistochemical (IHC) and immunofluorescence (IF) staining to determine the distribution of the expression of the ion transport genes *FOXI1*, *KCNMA1*, and *SLC12A2* in yak lung tissues. qRT-PCR and WB results indicated that mRNA and protein relative expression levels of *FOXI1* and *SLC12A2* were significantly higher in neonatal yaks than in juvenile and adult yaks (all *p* < 0.05), whereas those of *KCNMA1* were significantly higher in adult yaks than in neonatal and juvenile yaks (all *p* < 0.05). IHC and IF results demonstrated that FOXI1, KCNMA1, and SLC12A2 were distributed among the epithelial mucosal layers (including ciliated, goblet, and Clara cells) of the yaks’ bronchi and their branches in the lungs across different age groups of yak. Therefore, our results suggested that FOXI1, KCNMA1, and SLC12A2 may be strongly associated with the development and aging processes in yak lungs. These results provide insights into the molecular mechanisms underlying the yak’s adaptation to high-altitude environments and valuable references for further research.

## 1 Introduction

The yak (*Bos grunniens*) is a unique cattle breed native to cold, high-altitude pastoral areas of the Qinghai–Tibet Plateau. Compared with low-altitude yellow cattle, yaks possess different structural features such as a larger alveolar surface area, thinner alveolar spacing, and a thinner gas–blood barrier in the lung tissue. These characteristics allow yaks to adapt to the cold, high-altitude, and severely hypoxic environment of the Qinghai–Tibet Plateau. Therefore, yaks are considered an excellent model for studying mammalian high-altitude adaptation (1). Pulmonary fibrosis is a pathological condition characterized by abnormal fibrous connective tissue proliferation and deposition in the lung tissue, which results in impaired lung function (2). Ion transport is strongly associated with pulmonary fibrosis: ion channels and transport proteins regulate intracellular and extracellular ion balance, maintain cell membrane potential, and participate in cell signal transduction. This mechanism is regulated by pulmonary ionocytes, which are epithelial cells that strongly express cystic fibrosis (CF) transmembrane conductance regulator (CFTR) (3). In particular, pulmonary ionocytes can regulate the osmotic pressure, acidity, and viscosity of airway surface liquid and mucus. Loss-of-function mutations in CFTR in pulmonary ionocytes are associated with the development of CF, a genetic disorder (4). Continual exposure to a high-altitude, low-oxygen environment may lead to pulmonary fibrosis along with various pathological changes in the body. However, yak lungs rarely exhibit pathological manifestations such as hypoxia-induced pulmonary fibrosis. As such, the unique structure and specific regulation of ion transport in their lungs (5, 6) may allow yaks to effectively prevent abnormal changes induced by high-altitude, low-oxygen environments, including pulmonary fibrosis.

Ion transport proteins are crucial cellular regulatory factors, which are indispensable in the adaptive processes for high-altitude environments in yak lungs. The forkhead-box (FOX) family proteins play major roles in various tissues, including the brain, pancreas, lungs, and kidneys, during embryonic development (7). FOXI1 (Forkhead box transcriptional factor I1), a member of the FOX gene family, has been found to be expressed in mouse ectodermal structures, including the olfactory epithelium, oral epithelium, and the inner ear. In mice, FOXI1 mutations result in developmental defects in the inner ear tissue, leading to hearing and balance disorders (8). Brain studies have indicated that FOXI2 (Forkhead box transcriptional factor I2), expressed in the diencephalon, has a primary influence on forebrain development (9). FOXI2 is frequently expressed in epithelial structures, which are major sites for cell proliferation and differentiation, thus indicating its involvement in organismal development regulation (10). Ion transport provides a physiological basis for yak survival in low-oxygen, severely cold, and other extreme climatic conditions by regulating cell membrane potential and maintaining intracellular and extracellular ion balance. Ion channels and transport proteins also play a role in the functional regulation of immune cells, influencing inflammatory cell activity and intercellular signal transduction. This is crucial for the body’s defense against pathogen invasion and maintenance of immune balance in the respiratory system (11).

The yak has been used as an animal model for studying high-altitude hypoxia adaptability and its underlying mechanisms. However, an exploration into the relationship between ion transport–related functions in yak lungs and their adaptability to low-oxygen environments is lacking. In the current study, we used RNA sequencing (RNA-seq) to sequence the yak lung cell transcriptome at different stages of life. In particular, we aimed to identify differentially expressed genes (DEGs) and key pathways associated with yak lung development. Furthermore, we investigated the role of ion transport–related gene regulation in lung growth and development in yaks, speculating on their potential roles in yaks’ adaptation to high-altitude hypoxic environments. In this study, we provide crucial information that may enable subsequent in-depth research on low-oxygen adaptation mechanisms in yak lungs. Our results also offer valuable data enabling further exploration of the mechanisms underlying functional maintenance, unique evolutionary processes, and genetic mechanisms at the molecular level in yak lungs.

## 2 Materials and methods

### 2.1 Sample collection

Animal care and experimentation protocols were performed in accordance with the standards outlined in the *Guidelines for the Care and Use of Laboratory Animals in China*. All methods were approved by the Animal Ethics Committee of Gansu Agricultural University (Ethic approval file NO.GSAU-Eth-VMC-2022-23). Nine healthy yaks, free of obvious clinical disorders, were selected from Qingyuan Slaughterhouse in Linxia Hui autonomous prefecture, Gansu Province, China. The yaks were categorized into three age groups (*n* = 3 per group): 1 day (neonatal), 1 year (juvenile), and 4 years (adult). Following slaughter, lung tissue samples were promptly collected, rinsed with sterile physiological saline, and then placed in tissue preservation solution for transport to the laboratory within 2 h. At the laboratory, the samples were divided into two portions; of them, one was placed in 4% paraformaldehyde and then used for immunohistochemistry and immunofluorescence experiments, whereas the other was immediately frozen in liquid nitrogen and then used for quantitative real-time reverse-transcription polymerase chain reaction (qRT-PCR), and Western blotting, and sequencing.

### 2.2 RNA extraction and cDNA library generation

We extracted total RNA from the frozen yak lung tissue samples from each age group by using the TRIzol (Invitrogen, CA, United States) reagent. The extracted RNA samples were assessed for integrity and potential contamination through 1% agarose gel electrophoresis. RNA purity was determined on a NanoPhotometer spectrophotometer by measuring the OD260/280 and OD260/230 ratios. Moreover, RNA integrity was verified on an Agilent 2100 bioanalyzer, and rRNA was removed. Polyadenylated eukaryotic mRNA was then enriched using magnetic beads with Oligo(dT)s. Subsequently, the enriched polyadenylated mRNA was fragmented through ultrasonication. The fragmented mRNA was used as a template for double-stranded complementary DNA (cDNA) synthesis. The resulting polymerase chain reaction (PCR) products (i.e., the cDNA) were then subjected to selection, amplification, and purification by using AMPure XP beads. Finally, the purified cDNA was used to construct cDNA libraries. This process ensured. high-quality RNA extraction and polyadenylated mRNA enrichment, followed by generation of representative cDNA libraries for subsequent analyses. The results provided crucial insights into lung tissue transcriptomes across different age groups of yak.

### 2.3 RNA-seq and differential gene expression analysis

The amplified products were subjected to high-throughput sequencing on the Illumina HiSeq X Ten platform. Raw sequencing data were subjected to quality control assessment using FastQC, allowing for the filtration of low-quality data. Moreover, we used FastQC to assess the raw sequencing data, filter out low-quality data, and align the resulting clean data to the yak reference genome (Ensembl release 110) (12). In transcriptome sequencing analysis, we used fragments per kilobase of transcript per million mapped reads (FPKM) values to quantify gene expression levels. The R package DESeq2 was used for significant differential expression analysis across the samples. The criteria applied for filtering were *p* < 0.05 and |log_2_(fold change)| > 2, which resulted in DEG identification. Subsequently, the DEGs were subjected to Gene Ontology (GO) and Kyoto Encyclopedia of Genes and Genomes (KEGG) enrichment analysis by using ClusterProfiler.

### 2.4 DEG validation through qRT-PCR

To validate our RNA-seq data’s reliability, we randomly selected nine DEGs and subjected them to expression level verification through qRT-PCR. As presented in Table 1, qRT-PCR primers were designed using Primer (version 6.0; Premier Bio-soft International, Canada) and synthesized by Sangon Biotech (Shanghai, China). *ACTB* was used as the internal reference. cDNA was synthesized using an Evo M-MLV reverse transcription kit (Aikerui, China) according to the manufacturer’s instructions. The PCR system volume was 20 μL, and it comprised 10 μL of SYBR Green Mix, 8 μL of ddH_2_O, 1 μL of cDNA, and 0.5 μL each of the upstream and downstream primers. The reaction conditions were as follows: 95°C for 5 min, followed by 40 cycles of 95°C for 30 s, 61°C for 30 s, and 72°C for 10 s, and then, by 72°C for 2 min. For each gene, four replicates were analyzed (13). The relative expression of DEGs was analyzed using the 2^−ΔΔCT^ method, and the data were analyzed using analysis of variance (ANOVA) on SPSS (version 24.0).

**Table 1:** Primers for validation of DEGs.

Gene	Primer sequence (5′→3′)	Tm/°C	Length (bp)	GenBank No.
β-actin	F:CCGTGACATCAAGGAGAAG R:AGGAAGGAAGGCTGGAAG	61	174	XM_005887322.2
FOXP2	F:ATCGACAGCAATGGGAACAGCAG R:GGCAGTCTTCATCCTCGGCAATC	61	103	XM_005898908.1
TRPM2	F:ACCTACATTGACGGCGTGAACTTC R:TAGAGGCAGAGCAGAAGGACAGTC	61	146	XM_014482105.1
GABRA3	F:GTGAAGCAGGATATTGGCGGACTC R:CAGGTCGCAGCCGATTGTCATAG	61	133	XM_005888785.1
KCNC3	F:CTCATCTCCATCACCACCTTCTGC R:GGCTCCGTCTCCACCTCCAC	61	137	XM_005905037.1
ATP6V1B2	F:CCTCTCCCAGCCTCGTCTCAC R: ACTCCGCTTCGTCCCGTCAG	61	133	XM_005899927.2
ADGRF5	F:CAAGCCGAGCAAGCAGGAGAAG R:CCCCAGGTGAGCCCCAAGAG	61	87	XM_005910579.2
SLCA2A7	F:GTGGTCTTCGTGGGTGTCAAGTAC R:AGCGTAGATGGCGAGGATGGAG	61	87	XM_014477896.1
SLC26A4	F:ATCGCCTTCGGAATCAGCAACATC R:ACAGCGGTGCGGGACAGG	61	80	XM_005901425.1
SOX4	F:CGCAAGATCATGGAGCAGTCACC R:CTCCCTCCGCCTCCCGAATG	61	121	XM_005888411.2

### 2.5 mRNA expression determination through qRT-PCR

We selected genes encoding the key proteins involved in ion transport: *FOXI1*, *KCNMA1*, and *SLC12A2*. Primers for amplification were designed using Primer (version 6.0) and synthesized by Sangon Biotech. The primer sequences are provided in Table 2. The experimental procedures were consistent with those described in Section 2.4, aiming to detect the expression of these mRNA in various yak lung samples.

**Table 2:** Primers for determination of mRNA expression.

Gene name	Primer sequence (5→3)	Tm/°C	/bp	GenBank No.
β-actin	F:CCGTGACATCAAGGAGAAG R:AGGAAGGAAGGCTGGAAG	61	174	XM_005887322.2
FOXI1	F:GCAGAACTCCATCCGCCACAAC R:GGGGTCCAGGGTCCAGTAATTCC	61	103	XM_005911395.2
KCNMA1	F:CAGCGTTCGCCGTCAGTGTC R:CCAGGGTCCGTATCAGGGTAAGG	61	84	XM_005893787.1
SLC12A2	F:GCAACTGGCATTCTGGCTGGAG R:TCCCGAACAACACACGAACCTAC	61	149	XM_005888414.2

### 2.6 Western blotting

The yak lung tissue samples from different age groups were ground into a powder and subjected to protein extraction and denaturation. The denatured protein samples were subjected to 10% Sodium dodecyl sulfate polyacrylamide gel electrophoresis. The proteins were then transferred onto polyvinylidene fluoride membranes in a wet transfer system. After they were blocked with skimmed milk at room temperature for 4 h, the membranes were individually incubated with primary antibodies against FOXI1, SLC12A2, and KCNMA1 (each diluted at 1:800) at 4°C overnight. Next, the membranes were washed 10 times with PBST for 5 min, followed by incubation with secondary antibodies (goat antirabbit IgG, diluted at 1:3,000) on a shaker at room temperature for 1.5 h. After another round of 10 washings with PBST for 5 min, the membranes were treated with enhanced chemiluminescence reagent for visualization; next, the images were scanned using a chemiluminescence imager (13). Band intensity analysis was performed using ImageJ, and the target bands’ grayscale values were compared with those of β-actin bands. The relative expression levels of the target bands were calculated, and statistical analysis of the data was subjected to ANOVA on SPSS.

### 2.7 Immunohistochemical and immunofluorescence staining

After fixing them in 4% paraformaldehyde, we sliced the yak lung samples into approximately 1-cm^3^ blocks, subjected to dehydration in an alcohol gradient, clearing, embedding in paraffin, and finally, sectioned into 4 μm-thick sections.

We used the streptavidin peroxidase method to perform immunohistochemical staining of the prepared paraffin sections. In brief, deparaffinization and rehydration were performed in xylene and an alcohol gradient; this was followed by antigen retrieval, endogenous peroxidase blocking with a peroxide-blocking agent, and incubation at 37°C for 20 min. The sections were washed five times with phosphate-buffered saline (PBS) for 5 min and then incubated with a bovine serum albumin (BSA) blocking solution at room temperature for 15 min. The sections were incubated with primary antibodies against FOXI1 (DF8818, Affinity, America), SLC12A2 (DF2245, Affinity, America), and KCNMA1 (DF8570, Affinity, America) (diluted at 1:250, 1:300, and 1:300, respectively) at 4°C overnight; here, in the negative control, PBS was used instead of primary antibodies. Next, the sections were washed five times with PBS for 5 min. The sections were then exposed to biotin-labeled goat anti-rabbit IgG, followed by incubation at 37°C for 20 min and then by three washings with PBS for 5 min. Then, the sections were incubated with a horseradish peroxidase–labeled streptavidin working solution at 37°C for 20 min and then washed three times with PBS for 5 min. This was followed by DAB staining, hematoxylin staining, differentiation, bluing, dehydration, and transparency induction. Finally, the sections were sealed using a neutral resin, followed by microscopic observation of protein distribution in yak lung tissues (14).

For immunofluorescence staining, the paraffin sections were subjected to dewaxing, antigen retrieval, and blocking using the protocol used in the immunohistochemical staining method described above. The sections were incubated with primary antibodies against FOXI1, SLC12A2, and KCNMA1 (diluted at 1:300, 1:400, and 1:400, respectively) at 4°C overnight; here, in the negative control, we used PBS instead of primary antibodies. Next, the sections were washed five times with PBS for 5 min. The sections were then treated in the dark with fluorescent secondary antibodies (A32731, Thermo, America) and incubated at room temperature for 20 min. This was followed by 10 washings with PBS with Tween detergent (PBST) for 6 min. Nuclear staining with 4′,6-diamidino-2-phenylindole was performed at room temperature for 15 min, followed by five washings with PBST for 5 min. Finally, the sections were sealed and examined under a fluorescence microscope (15).

## 3 Results and analysis

### 3.1 Transcriptome data analysis

**Figure 1:**
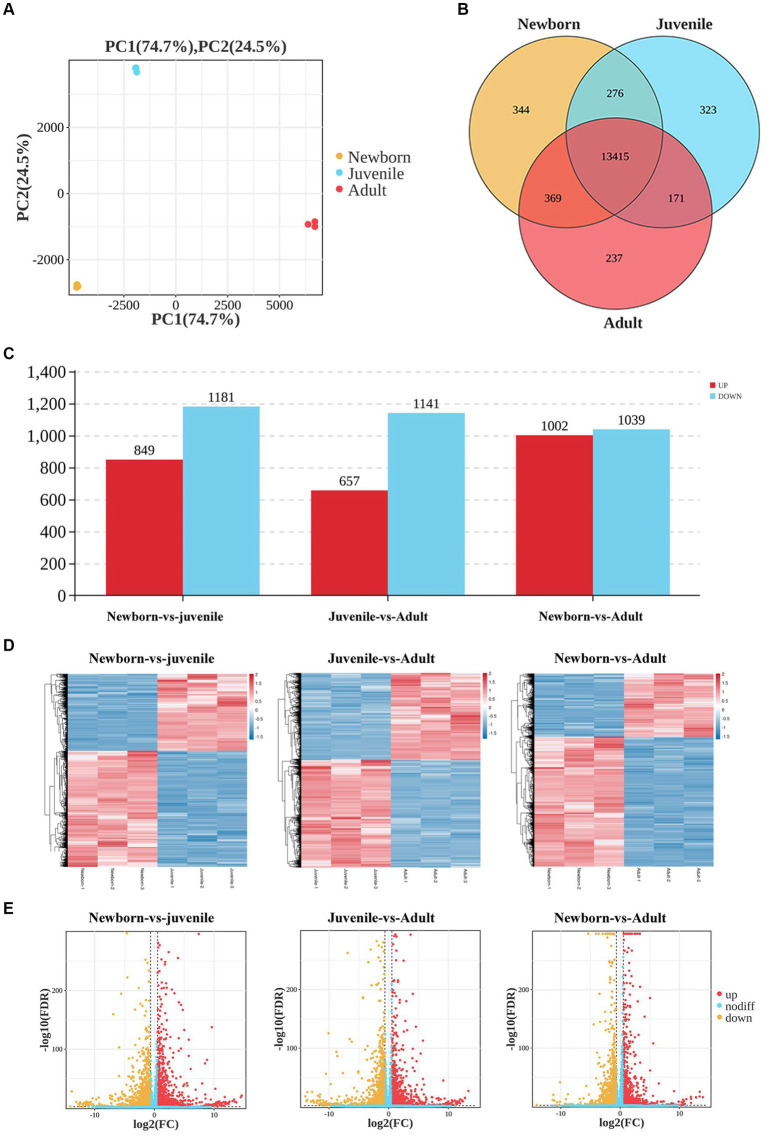
Analysis of DEGs in lung tissues from yaks of different ages. **(A)** Sample PCA plot, **(B)** venn diagram of DEGs, **(C)** histogram of DEGs, **(D)** heatmaps, and **(E)** volcano plots of DEGs in lung tissues from yaks of different ages.

We first used neonatal, juvenile, and adult yak lung tissue samples for transcriptome sequencing. The quantity of RNA samples exceeded 10 μg, with purity assessment (i.e., OD260 nm/OD280 nm) greater than 2.0, and RIN values exceeding 7, indicating successful quality control of all RNA samples, leading to the effective construction of nine libraries. Our principal component analysis (PCA) scatter plot (Figure 1A) demonstrated that samples from the three age groups were dispersed, exhibiting good intragroup sample clustering. This result suggested the reliability of our gene expression results obtained through sequencing, with a relatively similar gene expression profile among all samples.

We then generated Venn diagrams (Figure 1B) based on the number of genes with FPKM values of ≥1 in each age group. In the newborn, juvenile, and adult groups, 14,404, 14,185, and 14,192 genes were detected, respectively, with 344, 323, and 237 unique genes expressed in each group. Commonly expressed genes across the three age groups numbered 13,415. In our differential gene expression analysis Histogram (Figure 1C), by using DESeq2 with the criteria of false discovery rate < 0.05 and |log_2_(fold change)| > 2, we noted 2,030 DEGs (849 upregulated and 1,181 downregulated) between neonatal and juvenile yaks, 1,798 DEGs (657 upregulated and 1,141 downregulated genes) between neonatal and adult yaks, and 2,041 DEGs (1,002 upregulated and 1,039 downregulated) between juvenile and adult yaks. The heatmap (Figure 1D) and volcano plot (Figure 1E) were generated for DEG visualization, revealing a trend of well-defined overall gene expression.

### 3.2 GO and KEGG analysis of DEGs

**Figure 2:**
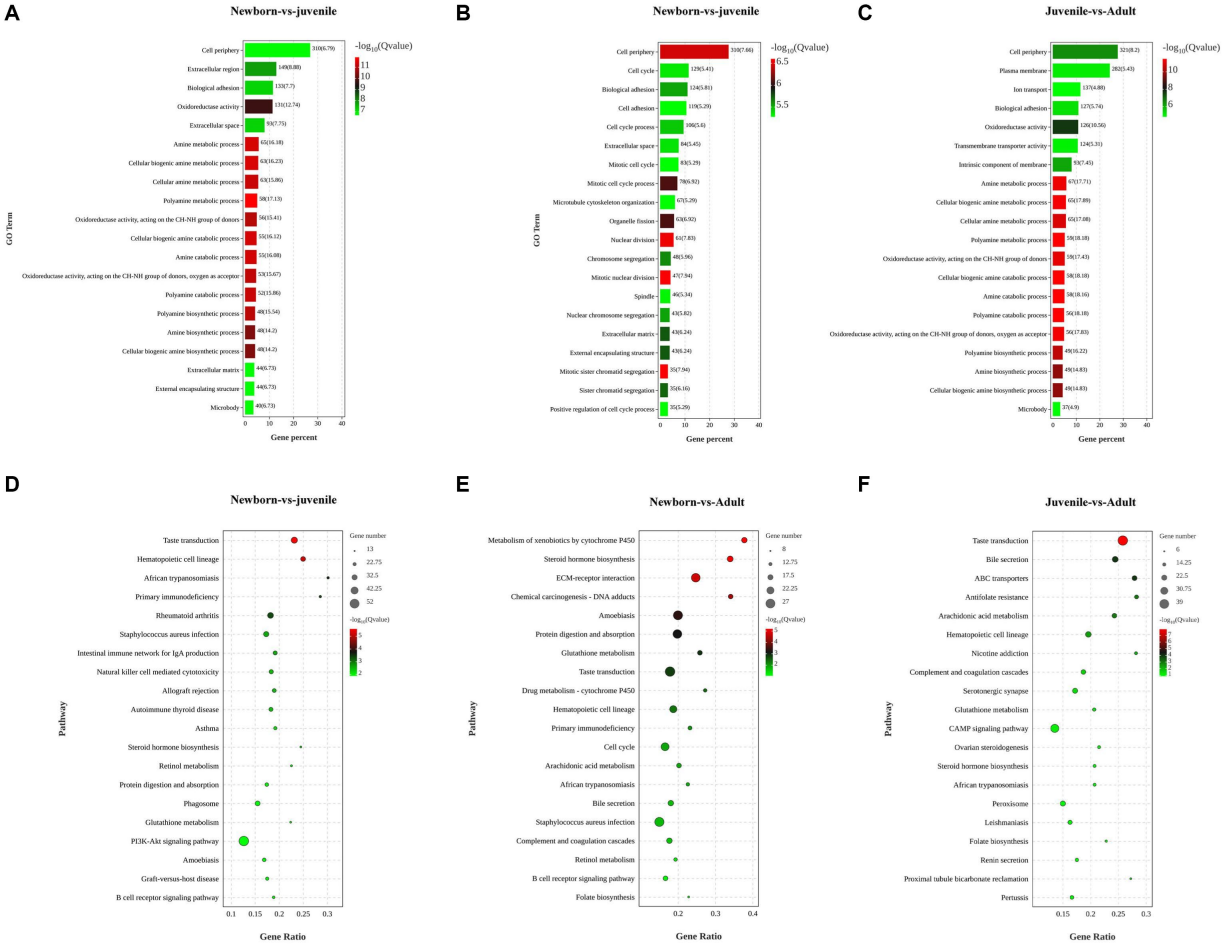
Functional enrichment analysis of DEGs in lung tissues from yaks of different ages. (A) GO and (D) KEGG analyses for DEGs among newborn and juvenile yaks. (B) GO and (E) KEGG analyses for DEGs among juvenile and adult yaks. (C) Differential GO and (F) differential KEGG analyses for DEGs among newborn and adult yaks.

To explore the biological functions of the DEGs, we performed GO and KEGG enrichment analyses through separate comparisons of DEGs in newborn yaks with those in juvenile yaks, those in juvenile yaks with those in adult yaks, and those in newborn yaks with those in adult yaks. The GO functional enrichment analysis between the neonatal and juvenile groups primarily focused on processes such as polyamine catabolic metabolism, cellular biogenic amine synthesis, and oxidoreductase activity (Figure 2A). The top 20 significantly enriched pathways for DEGs between these groups encompassed steroid hormone biosynthesis, retinol metabolism, protein digestion and absorption, PI3K-Akt signaling pathway, and B cell receptor signaling pathway (Figure 2D). For the juvenile versus adult groups, the GO analysis mainly targeted cellular biogenic amine catabolic process, intrinsic components of membranes, transmembrane transporter activity, and ion transport (Figure 2B), with the pathways chiefly involving bile secretion, ABC transporters, cAMP signaling pathway, and renin secretion in proximal tubule bicarbonate reclamation (Figure 2E). The GO analysis for the newborn versus adult groups was mainly concentrated on mitosis, cell adhesion, microtubule structure, and sister chromatid separation (Figure 2C), with the pathways primarily focusing on protein digestion and absorption, drug metabolism, arachidonic acid metabolism, bile secretion, and folate biosynthesis (Figure 2F).

### 3.3 GO and KEGG analysis of ion transport-related genes

**Figure 3:**
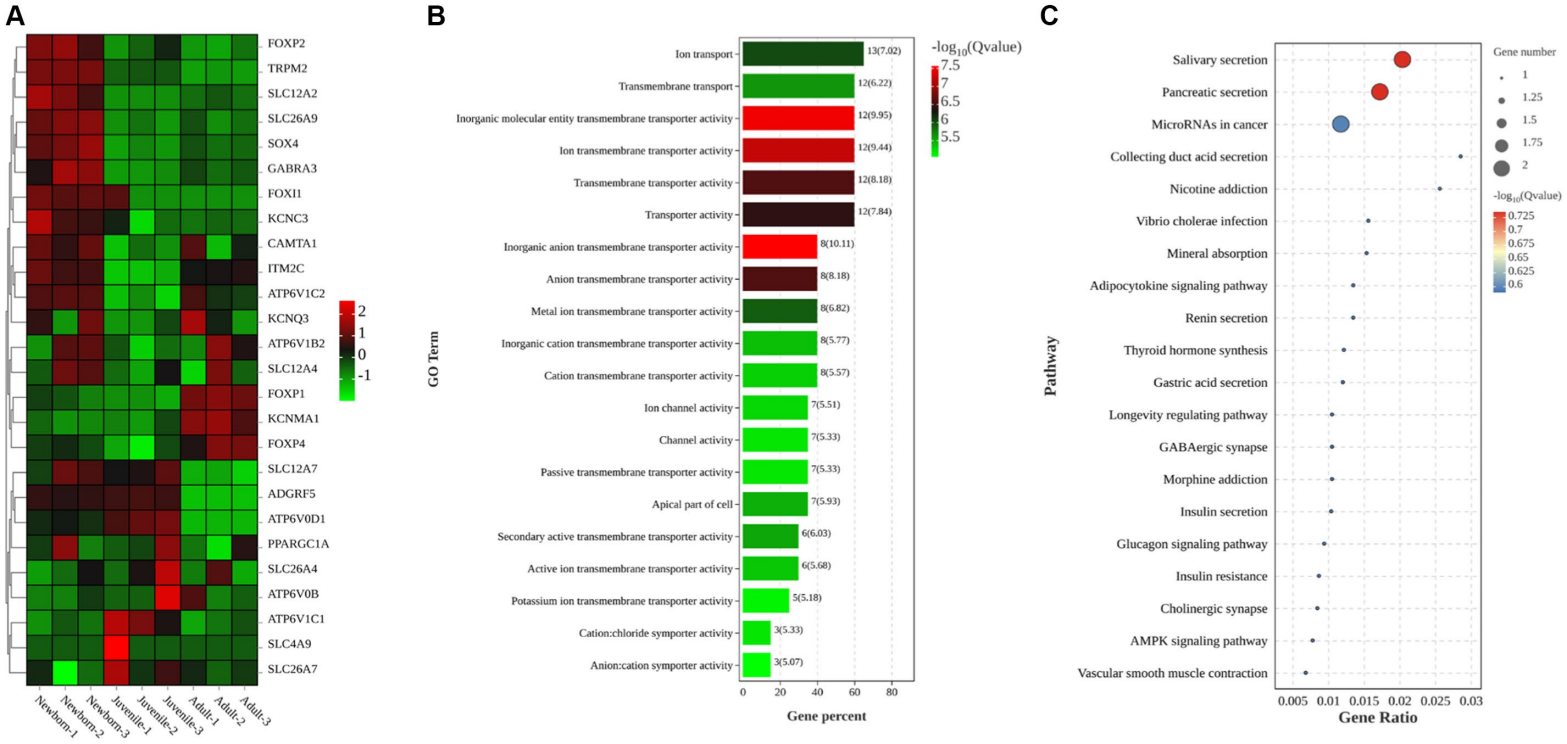
Clustering heatmaps and GO and KEGG enrichment analyses of target DEGs. **(A)** Clustering heatmap of target DEGs; red and green denote high and low expression, respectively. **(B)** GO enrichment analysis of DEGs **(C)** KEGG enrichment analysis of DEGs.

In this study, we primarily focused on subcategories under the categories “Cellular Component” (e.g., cell parts and biogenesis), “Molecular Function” (e.g., transporter activity), and “Biological Process” (e.g., cellular processes). After a comprehensive review of all relevant studies (regardless of their study location), we identified 26 DEGs related to ion transport (Figure 3A): *FOXP2*, *TRPM2*, *SLC12A2*, *SLC26A9*, *SOX4*, *GABRA3*, *FOXI1*, *KCNC3*, *CAMTA1*, *ITM2C*, *ATP6V1C2*, *KCNQ3*, *AP6V1B2*, *SLC12A4*, *FOXP1*, *KCNMA1*, *FOXP4*, *SLC12A7*, *ADGRF5*, *ATP6V0D1*, *PPARGC1A*, *SLC26A4*, *ATP6V0B*, *ATP6V1C1*, *SLC4A9*, and *SLC26A7*. Of them, 15 genes were upregulated, whereas 11 were downregulated. To further explore the biological functions of the selected DEGs, we performed GO enrichment analysis on the identified gene set and noted significant enrichment in 198 GO terms, including 136 biological processes, 21 cellular components, and 41 molecular functions. The histogram in Figure 3B displays the top 20 significantly enriched GO terms, with a focus on activities such as inorganic anion transmembrane transporter activity, transporter activity, ion transport activity, transmembrane transport activity, and apical cell activity. We also used KEGG enrichment analysis to elucidate the major biochemical metabolic and signaling pathways associated with the identified target genes. Figure 3C illustrates the top 20 significantly enriched pathways (*p* < 0.05), including salivary secretion, pancreatic secretion, cancer microRNA expression, duct acid secretion, adipocytokine signaling, renin secretion, thyroid hormone synthesis, gastric acid secretion, and insulin secretion. Therefore, these results demonstrated that the most prominently enriched KEGG pathways were the salivary and pancreatic secretion pathways.

### 3.4 Regulatory network analysis of DEGs

**Figure 4:**
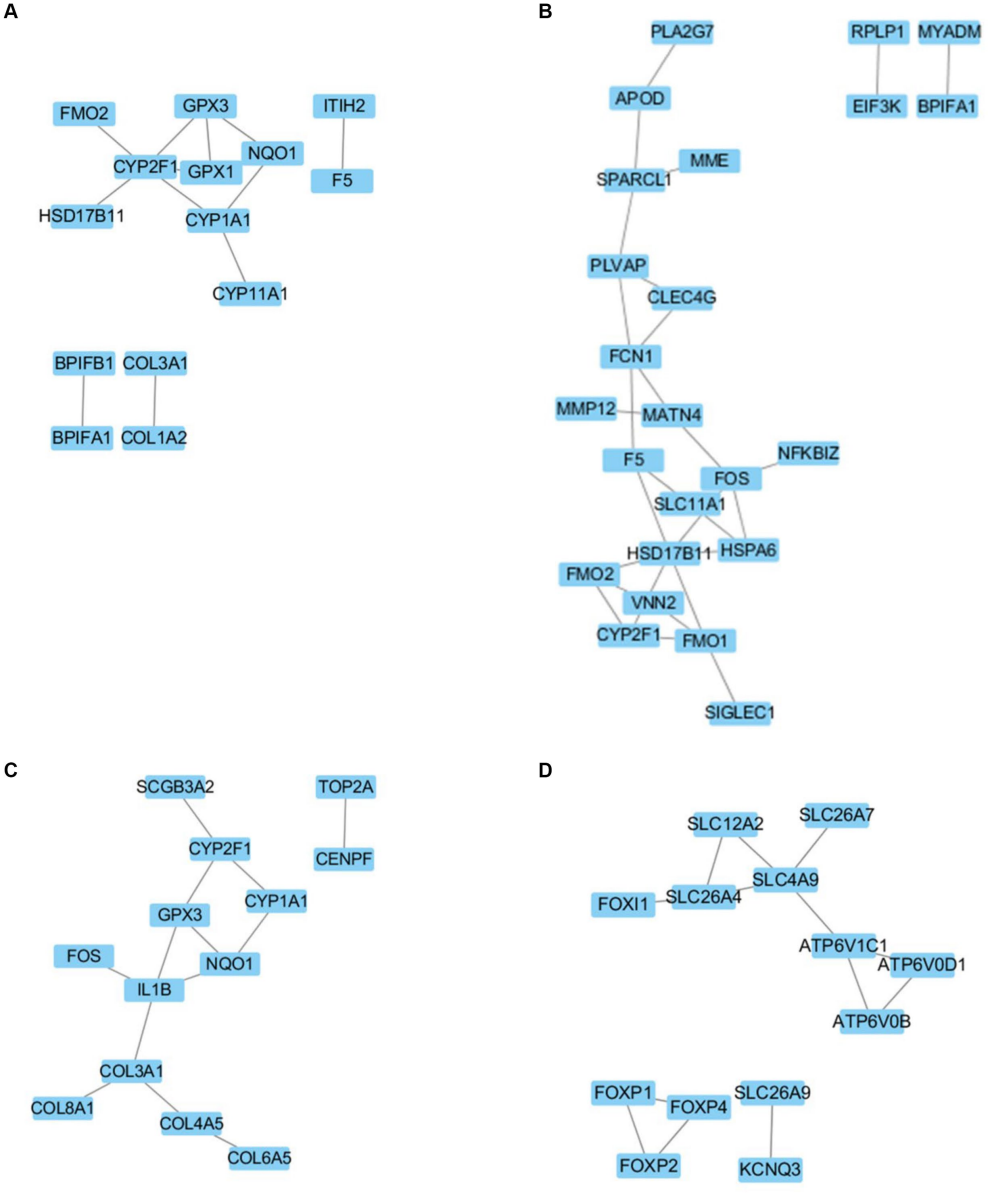
Regulatory network for **(A)** newborn and juvenile yaks, **(B)** juvenile and adult yaks, **(C)** newborn and adult yaks, and **(D)** the target gene.

In the comparison of DEGs between neonatal and juvenile yaks (Figure 4A), we noted that *CYP2F1* regulates *FMO2*, *HSD17B11*, *CYP1A1*, *GPX1*, and *GPX3*; *CYP1A1* regulates *CYP2F1*, *NQO1*, and *CYP11A1*; and *GPX3* regulates *CYP2F1*, *GPX1*, and *NQO1*. In the comparison of DEGs between juvenile and adult yaks (Figure 4B), we observed that *FCN1* regulates *PLVAP*, *CLEC4G*, *MATN4*, and *F5*; *HSD17B11* regulates *F5*, *SLC11A1*, *HSPA6*, *FMO1*, *VNN2*, and *FMO2*; and *FOS* regulates *MATN4*, *NFKBIZ*, *HSPA6*, and *SLC11A1*. In the comparison of DEGs between neonatal and adult yaks (Figure 4C), we noted that *CYP2F1* regulates *SCGB3A2*, *GPX3*, and *CYP1A1*; IL1B regulates *GPX3*, *NQO1*, *COL3A1*, and *FOS*; and *COL3A1* regulates *IL1B*, *COL4A5*, and *COL8A1*. Finally, our comparisons within the ion transport–related target gene network (Figure 4D) demonstrated that *SLC26A4* regulates *FOXI1*, *SLC12A2*, and *SLC4A9*; *SLC4A9* regulates *SLC12A2*, *SLC26A7*, *ATP6V1C1*, and *SLC26A4*; *ATP6V1C1* regulates *SLC4A9*, *ATP6V0D1*, and *ATP6V0B*; and *FOXP2* regulates *FOXP1* and *FOXP4*.

### 3.5 DEG validation through qRT-PCR

**Figure 5:**
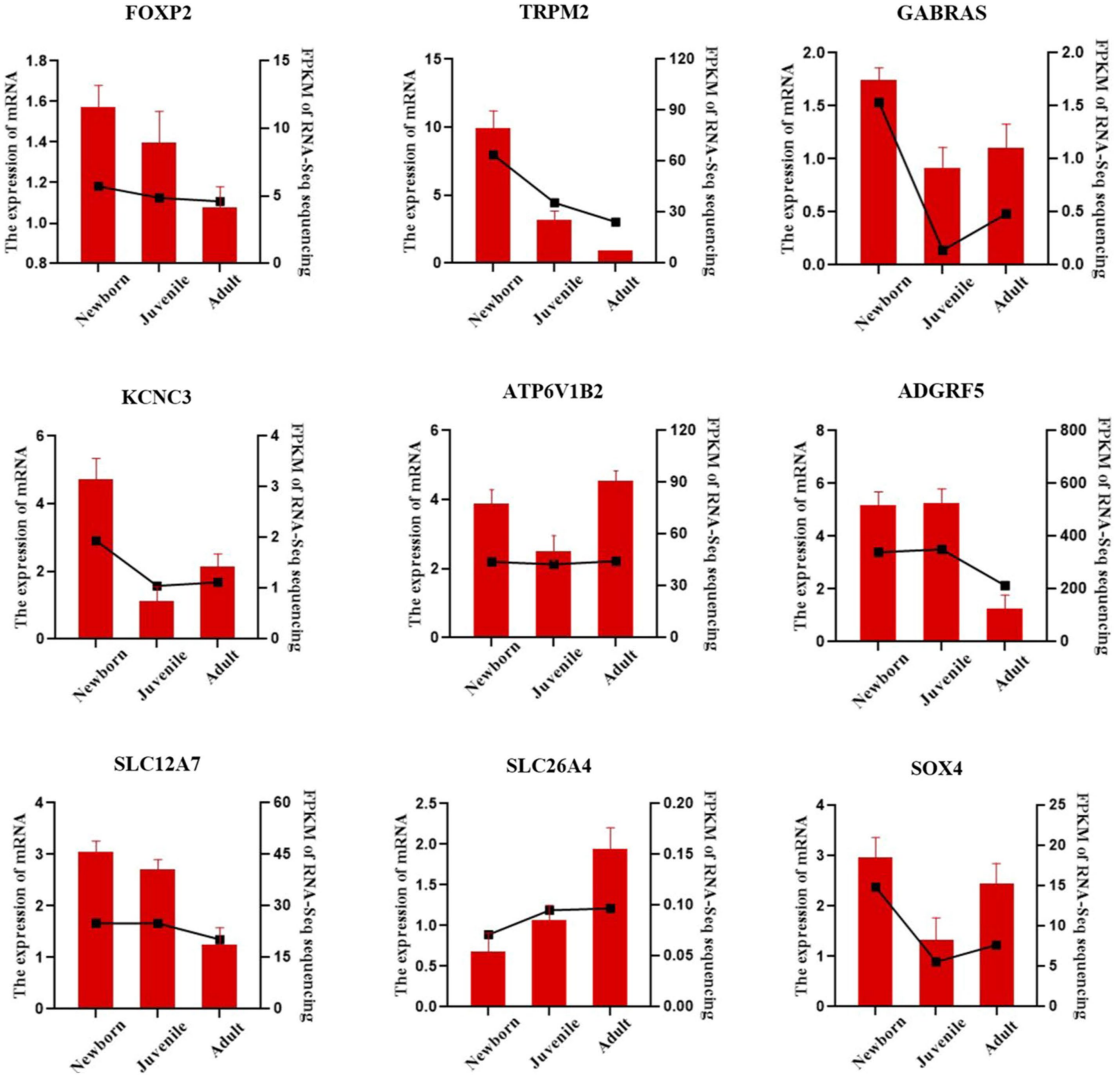
qRT-PCR verification of lung DEGs in yaks. The bar chart presents our qRT-PCR verification results, whereas the dashed line chart denotes our RNA-seq results.

We validated our bulk RNA-seq results in yak lung through qRT-PCR methodology. We selected the nine DEGs *FOXP2*, *TRPM2*, *GABRA3*, *KCNC3*, *ATP6V1B2*, *ADGRF5*, *SLC12A7*, *SLC26A4*, and *SOX4*, were from the identified set. *ACTB* was employed as the internal reference gene. As illustrated in Figure 5, the qPCR validation results exhibited a trend similar to the FPKM values from RNA-seq, indicating the reliability of the transcriptome sequencing data for subsequent analyses.

### 3.6 qRT-PCR and Western blotting for verification of *FOXI1*, *KCNMA1*, and *SLC12A2* expression

**Figure 6:**
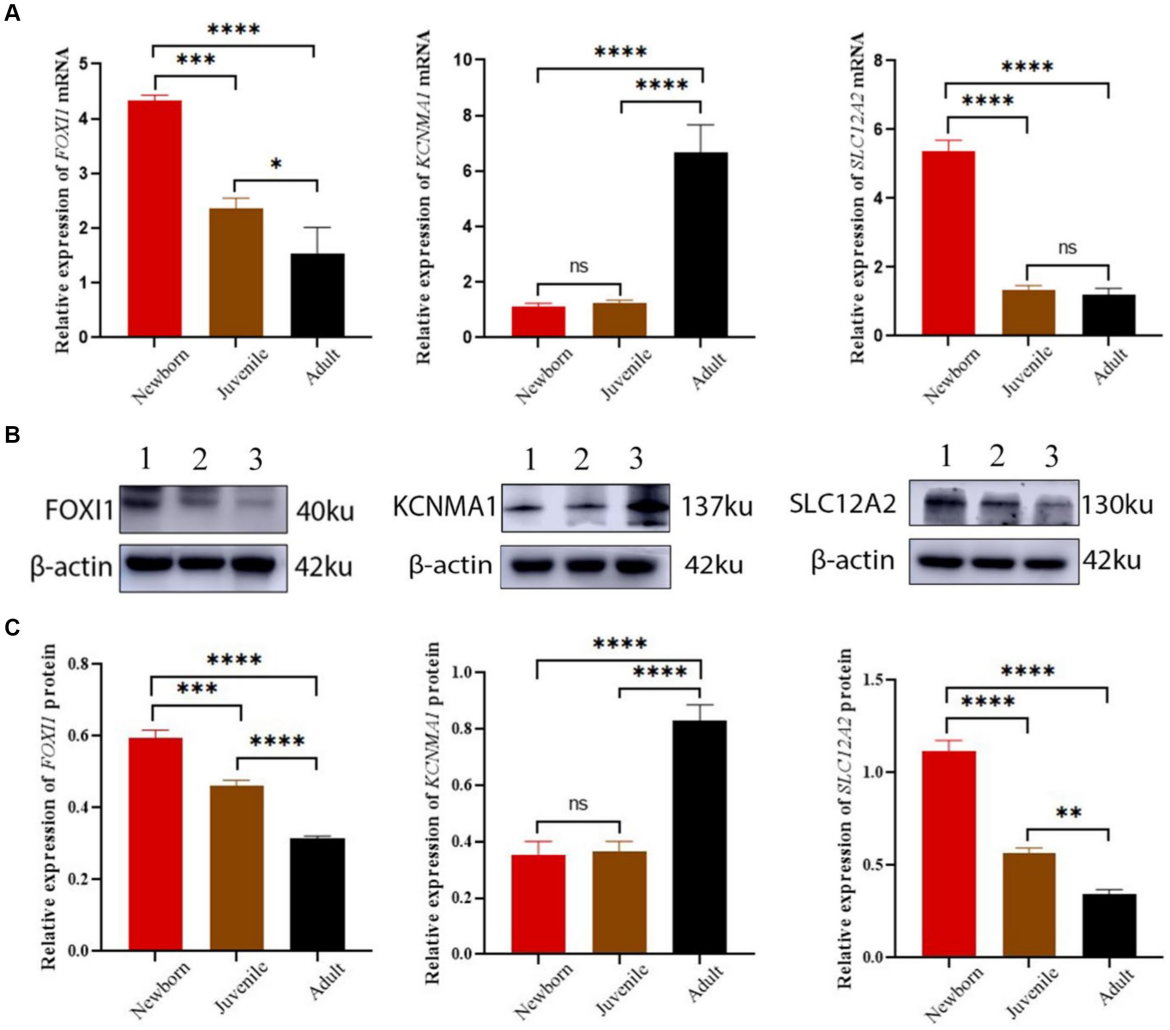
*FOXI1*, *KCNMA1*, and *SLC12A2* mRNA and protein expression in lungs of yaks of different ages. **(A)** *FOXI1*, *KCNMA1*, and *SLC12A2* mRNA expression results. **(B)** Western blots for FOXI1, KCNMA1, and SLC12A2. **(C)** Western blotting results of FOXI1, KCNMA1, and SLC12A2 expression. 1, 2, and 3 denote newborn, juvenile, and adult yaks, respectively. *significant (*p* < 0.05); ns, nonsignificant (*p* > 0.05).

Our qRT-PCR results demonstrated differential expression of *FOXI1*, *KCNMA1*, and *SLC12A2* among the lung samples from neonatal, juvenile, and adult yaks (Figure 6A). The relative *FOXI1* mRNA expression was the highest in neonatal yaks, followed by that in juvenile yaks; it was the lowest in adult yaks; all differences among age groups were significant (all *p* < 0.05). Relative *SLC12A2* expression was significantly higher in neonatal yaks than in juvenile and adult yaks (all *p* < 0.05). *FOXI1* and *SLC12A2* exhibited similar expression trends: a decrease with age. *KCNMA1* demonstrated a significantly higher relative expression in adult yaks than in neonatal and juvenile yaks (all *p* < 0.05); in general, *KCNMA1* expression increased with age.

We assessed FOXI1, KCNMA1, and SLC12A2 expression in neonatal, juvenile, and adult yak lung tissues through Western blotting. As shown in Figure 6B,C, all three proteins were expressed in yak lung tissues at different ages. In particular, FOXI1 and SLC12A2 exhibited the highest relative expression levels in neonatal yaks, followed by juvenile yaks; however, FOXI1 and SLC12A2 expression was the lowest expression in adult yaks. The expression levels of these genes decreased with age, and significant differences were observed among age groups (all *p* < 0.05). *KCNMA1* demonstrated a significantly higher relative expression level in adult yaks than in neonatal and juvenile yaks (all *p* < 0.05). Here, the expression levels increased with age.

In general, the verification results based on mRNA and protein expression levels indicated that lung *FOXI1*, *KCNMA1*, and *SLC12A2* mRNA and protein expression levels demonstrate a consistent trend of variations in yaks across different age groups.

### 3.7 Immunohistochemical and immunofluorescence staining for FOXI1, KCNMA1, and SLC12A2 expression distribution

**Figure 7:**
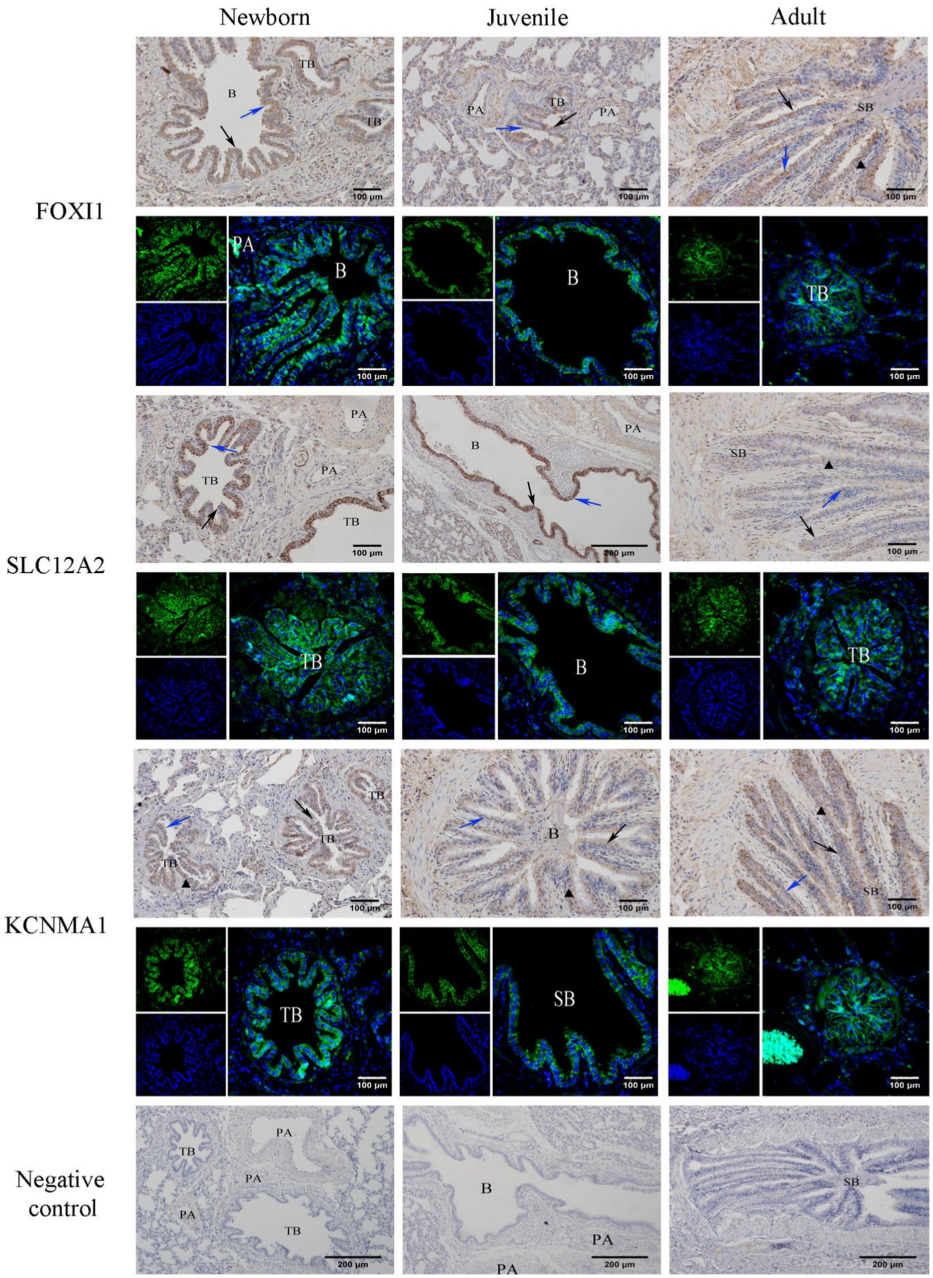
Immunohistochemical and immunofluorescence staining for FOXI1, KCNMA1, and SLC12A2. SB, B, TB, PA, ↑, ↑, and ▲ denote small bronchiole, bronchiole, terminal bronchiole, pulmonary artery, ciliated cell, Clara cell, and goblet cell, respectively.

Our immunohistochemical and immunofluorescence staining results (Figure 7) demonstrated that FOXI1, KCNMA1, and SLC12A2 exhibited a generally consistent distribution pattern in the lungs of yaks of different ages. They were evenly distributed in various bronchi, primarily in the epithelial mucosal layers (including ciliated, goblet, and Clara cells) of the yaks’ bronchi and their branches. In the terminal bronchioles, expression was observed in ciliated and Clara cells. Intergroup comparisons revealed that FOXI1 and SLC12A2 demonstrated stronger, positive expression in neonatal yaks compared with that in juvenile and adult yaks. By contrast, KCNMA1 exhibited stronger positive expression in adult yaks than in neonatal and juvenile yaks.

## 4 Discussion

In this study, we used RNA-seq-based transcriptome sequencing to elucidate the DEGs in the growing lung tissues of yaks over different age groups. In particular, transcriptome data from neonatal, juvenile, and adult yak lung tissues were analyzed to identify and functionally characterize DEGs. In total, 26 genes related to ion transport were identified. The current results provide reliable data for the functional exploration of ion transport–related genes in yak lungs and led to an improved understanding of the high-altitude environment–adaptive mechanisms in yaks at different ages.

Through GO enrichment analysis of DEGs in neonatal, juvenile, and adult yaks, we noted that the main enriched processes included amine catabolic processes and oxidoreductase activity. Amine catabolic processes regulate the levels of various neurotransmitters such as adrenaline, histamine, and dopamine, influencing neural signal transmission and respiratory system function. Histamine release may lead to vasodilation and increased permeability, triggering inflammatory reactions (16). Through the regulation of histamine levels via amine catabolism, the body can expedite immune system responses, facilitating the control of the inflammatory process. The ion transport function in the lungs plays a role in the regulation of the body’s acid–base balance. In particular, amine metabolites may affect the acid–base balance of the bodily fluids. In the lungs, amine metabolism regulation can aid in maintaining normal physiological acid–base balance, ensuring the normal operation of cells and tissues. Oxidoreductases, including xanthine dehydrogenase (XDH)/xanthine oxidase and NAD(P)H oxidase, operate within the mitochondria, participating in oxygen reduction reactions in the respiratory chain within cells. This process generates energy, providing power for the normal functioning of lung cells. By facilitating uric acid production, XDH contributes to cellular antioxidant defense, whereas the superoxide generated by NAD(P)H oxidase plays a role in immune cell activation, and therefore, pathogen clearance (17). This natural immune defense mechanism, strongly associated with the antioxidant process, aids yaks in adapting to cold, low-oxygen environments.

Our results revealed that the main signaling pathways in the lungs of yaks of different ages include the ABC transporter, PI3K-Akt, and AMP pathways. ABC transporters, ubiquitous proteins found in bacterial and eukaryotic cells, represent one of the largest protein families. Each member contains two highly conserved ABC proteins, which undergo dimerization on ATP binding and disassemble after ATP hydrolysis. Through conformational changes, ABC transporters transfer various substrates such as ions, sugars, lipids, peptides, proteins, and drugs across cell membranes (18). In the lungs of yaks of different ages, ABC transporters tend to demonstrate gradual maturation and enhancement and involve aspects such as drug metabolism, airway mucus clearance, and immune regulation.

The PI3K-Akt pathway primarily facilitates cell proliferation, inhibits apoptosis, and regulates tissue inflammation activated by cellular stimuli or toxic damage. The PI3K-Akt pathway may prevent cell apoptosis in the neonatal yak lungs, promoting alveolar and airway cell proliferation and development, thereby influencing lung structure formation and development. The immunoregulatory function of the PI3K-AKT pathway is mainly mediated through various receptors, including insulin receptors, pathogen-associated molecular pattern receptors, cytokine receptors, and hormones, participating in macrophage response regulation (19). In adult yak lungs, the PI3K-Akt pathway may be involved in responses to external environmental pressures, such as oxygen concentration changes or other environmental stimuli, to maintain lung adaptability and stability. The PI3K-Akt pathway may play a crucial regulatory role in the lungs of yaks of different ages, with specific functions varying during lung development and adaptation, ensuring adaptability to physiological and environmental changes. The cAMP pathway is a crucial intracellular signal transduction pathway involved in regulating various physiological processes such as transcription, translation, development, and proliferation.

cAMP, one of the most common second messengers, is activated by adenylyl cyclase (AC) on the interaction of ligands (mainly signaling molecules) with G protein-coupled receptors. cAMP directly acts on three major targets: protein kinase A, cAMP-activated exchange protein, and cyclic nucleotide-gated ion channels (20). Elevated intracellular cAMP levels can facilitate the alleviation of inflammation and airway fibrosis (21). We speculate that the cAMP pathway regulates various aspects of lung growth, development, and adaptation to low-oxygen environments in yaks.

Our results demonstrated signaling pathways related to ion transport, primarily related to the salivary and pancreatic secretion pathways. In the salivary secretion pathway, phospholipase C activation is triggered through parasympathetic nerve stimulation, leading to an increase in intracellular Ca^2+^ concentration. This process is involved in regulating fluid secretion, including water and ion transport (22, 23). In this pathway, *KCNMA1* and *SLC12A2* primarily contribute to the transport of Ca^2+^, Cl^−^, and HCO_3_^−^, maintaining a normal physiological environment. The pancreatic secretion pathway involves pancreatic duct cells secreting fluids and HCO_3_^−^. CFTR Cl^−^ channels and CFTR-dependent Cl^−^/HCO_3_^−^ ion exchange channels induced by cAMP are responsible for HCO_3_^−^ secretion (24). The pathways enriched in the KEGG enrichment analysis mainly function in various ions’ secretion and transport—aligning with the role of ion transport in maintaining ion balance within and outside cells and fluid homeostasis in the lungs. However, specific mechanisms underlying these functions warrant further investigation.

FOXI1, a transcription factor, plays a crucial regulatory role in ion transport and is essential for not only the development of many diseases but also embryonic development and cell differentiation. Exposure to *FOXI1* mRNA in developing embryos, such as in those of African clawed frogs, results in the upregulation of ionocyte genes, including those encoding vacuolar ATPase (V-ATPase) subunits in the embryonic epidermis (25). In contrast, *FOXI1* knockout leads to the loss of V-ATPase expression. Moreover, *Foxi1*-knockout mice lack pendrin (*Slc26A4*) expression, causing a defect in chloride ion reabsorption and resulting in ionocyte abnormalities (3). In ferrets, FOXI1 transcription activation leads to an increase in the expression of ionocyte genes, including *CFTR*, *ASCL3*, and *ATP6V0D2* (a gene encoding a V-ATPase subunit) (25). On the basis of data from different species and tissue environments, the aberrant expression of or mutations in ionocytes *FOXI1* may be associated with ion channel–related diseases. Notably, lung ionocytes represent a novel type of airway epithelial cell with high CFTR expression (3, 4). This result further confirms the significance of FOXI1 in the lungs in maintaining the ion balance in the airways. FOXI1 regulation plays a key role in maintaining CFTR expression, ensuring normal ion transport and thereby preserving the physiological function of the lungs (26). In this study, FOXI1 expression was found to become increasingly downregulated with age in yaks; in other words, FOXI1 expression was the highest in the lungs of neonatal yaks. Therefore, FOXI1 may be involved in ionocyte differentiation and ion transport protein expression.

The FOXP subfamily comprises four transcription factors (i.e., FOXP1, FOXP2, FOXP3, and FOXP4), which play crucial roles in various developmental processes. FOXP1 and FOXP4 loss leads to embryonic death due to defects in heart and lung development. In a study, mice lacking FOXP2 expression died approximately 3 weeks after birth due to motor disorders, indicating the importance of all FOXP proteins in normal development (27). In the current study, FOXP1 and FOXP4 expression was upregulated significantly; therefore, these transcription factors potentially contribute to the maintenance of tissue homeostasis, particularly in terms of cell differentiation, function, and metabolism, in adult yaks. In contrast, FOXP2 expression was downregulated, with its high expression in neonatal yaks, suggesting its major involvement in embryonic development (28). The FOXP family proteins were also found to regulate KCNC3, KCNQ3, SLC12A4, SLC4A9, and TRPM2. KCNC3, KCNQ3, and KCNMA1 are voltage-gated potassium ion channel proteins, and the downregulation of KCNC3 and KCNQ3 in yak lungs suggested that the expression of voltage-gated potassium ion channels decreases in yaks with age, reducing membrane depolarization and harmful effects of pulmonary edema (29, 30). KCNMA1 upregulation might be associated with its role in promoting vascular endothelial cell growth (31, 32). SLC12A2, SLC12A4, SLC12A7, SLC4A9, SLC26A7, SLC26A4, and SLC26A9, which are SLC12A, SLC4A, and SLC26A family proteins, are cation-coupled chloride cotransporter proteins. They are mainly responsible for the ion transport of Cl^−^ and HCO_3_^−^, ensuring strict regulation of cellular solutes and the extracellular environment, playing crucial roles in the maintenance of acid–base balance and fetal development (33, 34). Our results indicated an overall downregulation trend in these genes’ expression levels, suggesting that the downregulation is associated with enhanced environmental adaptation and increased resilience with age in yaks, enabling improved homeostasis maintenance.

ATP6V1C2, ATP6V1B2, ATP6V0D1, ATP6V0B, and ATP6V1C1 are the subunits of V-ATPase, a proton pump present on the cell membrane, which depends on ATP hydrolysis for its operation. Its primary function is proton transport, playing a crucial role in the regulation of cellular pH homeostasis, membrane trafficking, bone resorption, and defense against toxin invasion. The current results revealed an overall upregulation trend of V-ATPase in yak lungs, with slight variations possibly attributable to the specific subunit isoforms used for distinct physiological functions in different cell membranes. During yak growth and development, V-ATPase activity and expression increase gradually, potentially contributing to the maintenance of ion and acid–base balance inside and outside the lung cells; this also ensures a normal physiological environment fulfilling the growth and developmental needs of yak lungs at different life stages (35). With age, the lungs may require increasing amounts of energy and resources to support their growth, and V-ATPase upregulation might be an adaptation to meet this demand.

The current results also revealed that several key genes (*ADGRF5*, *GABRA3*, *SOX4*, *CAMTA1*, and *PPARGC1A*) became increasingly downregulated with age in yak lungs. These results suggested that during the neonatal stage, the aforementioned genes play a key role in adapting to the challenges of high-altitude, low-oxygen environments. With age, the expression levels of these genes decrease gradually, reflecting the progressive refinement of overall physiological functions and an enhancement in adaptability to low-oxygen environments. *ADGRF5* may be involved in processes related to cell adhesion and signal transduction. In the early stages of development, its upregulation may be related to the interaction between yak lung cells in a low-oxygen environment. *ADGRF5* encodes a V-ATPase regulator in lung ionocytes (25), and *ADGRF5* loss in renal cells has been noted to lead to the predominant loss of H^+^ in urine, causing mild metabolic alkalosis due to the reabsorption of HCO_3_^−^ in renal tubules (36). *ADGRF5* upregulation in the early stages of development may be associated with cell interactions in yak lungs under low-oxygen conditions.

The calcium channel α3 subunit GABRA3 has ATP- and protein kinase C-binding activities; it participates in various processes, including negative regulation of nitrogen compound metabolic processes, as well as positive regulation of glucose input and nitrogen compound metabolic processes. It plays an upstream or intracellular role in a cell’s response to insulin stimulation and positive regulation of sodium ion transport. Moreover, intraperitoneal injection of a certain amount of GABRA3 in mice significantly reduces oxygen consumption, enhances hypoxia tolerance, and prolongs their survival time under hypoxia (37). Moreover, GABRA3 participates in adaptive protection of the auditory nerve in high-altitude, low-oxygen environments, suggesting its regulatory role under hypoxic conditions (38). Being a gene associated with neurotransmission, *GABRA3*’s downregulation may indicate an increase in neural signaling among neurons, possibly in response to environmental stimuli, during the early stages of yak development. CAMTA1 may be related to stress responses, and its downregulation suggests a reduced sensitivity to low-oxygen stress during the aging process (39, 40). Peroxisome proliferator-activated receptor gamma coactivator 1α (PPARGC1A) is a key factor involved in energy metabolism, playing a biological role in adaptive thermogenesis, mitochondrial biogenesis, lipid synthesis, and gluconeogenesis, among other pathways (41, 42). PPARGC1A downregulation during the early stages may reflect adjustments in energy metabolism in response to low-oxygen environments. In general, changes in the expression of all the aforementioned key genes may be part of the biological responses of yaks to high-altitude environments. The gradual improvement of overall physiological functions with age enhances the yaks’ adaptability to low-oxygen environments.

In this study, we also performed immunohistochemical and immunofluorescence staining of the lung tissues from yaks of different ages for localizing the expression of three key ion transport factors, FOXI1, KCNMA1, and SLC12A2, within the lung tissues. The results demonstrated that all three ion transport factors were expressed in the mucosal epithelium of the bronchi and its branches, and their localization did not appear to change with age. Thus, the main sites for ion transport function in yak lungs may be located in the epithelial cells in the airway mucosal layer; moreover, aging may not influence the lung ionocytes’ distribution. We speculate that lung ionocytes may be primarily distributed in the epithelial cells of the airway mucosa. Moreover, lung ionocytes may share some functions with lung epithelial cells such as ciliated and Clara cells. These results further improve the current understanding regarding the correlation among different lung cell types. We also noted that neonatal yaks demonstrated the highest FOXI1 and SLC12A2 expression; this expression gradually decreased with age. In contrast, KCNMA1 demonstrated significantly higher expression in adult yaks compared with neonatal or juvenile yaks.

Changes in salt concentration in the environment can alter CFTR expression in zebrafish ionocytes (43). Therefore, we assessed our yak lung tissue samples for similar changes in the airways. During gas inhalation, sudden changes in tension in the airways may lead to pulmonary edema. Moreover, exposure to low-oxygen environments can lead to pulmonary fibrosis, primarily due to the thick mucus obstructing the airways and promoting inflammatory responses triggered by pathogen toxins. The primary function of pulmonary ionocytes is the regulation of the normal acid–base balance and viscosity of airway mucus. In the high-altitude, low-oxygen environments where yaks reside, maintaining the functional integrity of the CFTR protein is crucial to prevent the development of cystic fibrosis in the lungs, a risk heightened by hypoxia. Previous investigations by our research group have demonstrated that neonatal yaks exhibit significantly higher levels of CFTR protein expression compared to juveniles and adults, highlighting the pronounced effect of hypoxia on the neonatal stage and underscoring the pivotal role of CFTR in the neonatal adaptation to hypoxia and in mitigating pulmonary fibrosis. Additionally, the transcription factor FOXI1, a critical regulator of CFTR, shows elevated expression in the pulmonary tissues of neonatal yaks, indicating its primary role during the early life phase, which is most vulnerable to hypoxic stress. This elevated expression of FOXI1 is vital for cell development and differentiation, and it plays a significant role in the regulation of ion transport proteins like CFTR, SLC12A2, and KCNMA1, which are key to sustaining normal pulmonary physiology, preventing fibrotic lung changes, and facilitating adaptation to hypoxic conditions. We hypothesize that because they are less developed in various physiological aspects than adult yaks, newborn yaks are more susceptible to the effects of low oxygen levels, particularly in the lungs, where the primary function is gas exchange to support normal growth and development.

SLC12A2 and KCNMA1 are responsible for the transport of Na^+^, K^+^, and Cl^−^, which facilitates the maintenance of the essential ion exchange functions of pulmonary ionocytes (44, 45). Therefore, these proteins may have a crucial role in adaptation to high-altitude, low-oxygen environments, as well as in airway damage prevention and repair, in yaks. However, their precise mechanisms of action warrant further in-depth research. Nevertheless, our findings expand the current understanding of pulmonary ion transport and its role in the adaptation of yaks to high-altitude, low-oxygen environments.

This study revealed unique biological characteristics of yak lungs; these characteristics aid yaks in adapting to high-altitude, low-oxygen environments. With a thorough understanding of these key genes, the mechanisms underlying the rarely occurring hypoxia-induced pulmonary fibrosis in yaks at high altitudes can be elucidated. Moreover, our results serve as a reference for the adaptive biology research of other plateau-dwelling animals. Furthermore, our sequencing data may afford new research perspectives for future studies on related disorders.

## 5 Conclusion

Our RNA-seq results facilitated the identification of age-related DEGs and crucial pathways in yak lungs. Among them, *FOXI1*, *KCNMA1*, and *SLC12A2* were found to be distributed in the epithelial mucosal layers (including ciliated, goblet, and Clara cells) of the yaks’ bronchi and their branches. The expression patterns of these genes’ mRNA and proteins are consistent. The age-related differences in the expression of these genes are associated with the adaptive structural formation of yak lungs to low-oxygen environments and the maintenance of airway acid–base balance and the prevention of pulmonary fibrosis disease.

## Data availability statement

The original contributions presented in the study are publicly available. This data can be found at: https://www.ncbi.nlm.nih.gov/; PRJNA1071804.

## Ethics statement

The animal studies were approved by Ethic approval file No. GSAU-Eth-VMC-2022-23. The studies were conducted in accordance with the local legislation and institutional requirements. Written informed consent was obtained from the owners for the participation of their animals in this study.

## Author contributions

XX: Data curation, Writing – original draft, Writing – review & editing. YW: Writing – original draft. YC: Data curation, Writing – review & editing. QZ: Writing – original draft. HL: Data curation, Writing – original draft. LC: Data curation, Writing – original draft. JH: Data curation, Supervision, Writing – original draft.
